# Ethics—Professional

**Published:** 1898-01

**Authors:** H. E. Harlan


### Ethics.—Professional.

BY H. E. HARLAN, D.D.S.

Read before the Toledo Dental Society, November 12th, 1897.

Undoubtedly all have given more or less attention to the subject of advertising our business, and have come to some conclusion as to what is best and right, as to the manner in which we inform the public of our place of business, and of our special claims to prominence and superiority over our fellow-practitioners. That we all want business, and desire that the public shall recognize our ability to serve them skillfully, and that we all want compensation for our time, services and knowledge, I am presuming, is tacitly admitted and acknowledged by all.

The profession has presumed to establish a code of ethics that seems to me to be somewhat loose, and easy of interpretation to a few, and to others rigid and uncompromising, and having had some experience in advertising, both by printer's ink and otherwise, I have dared to offer you a few thoughts on the subject. I suppose that a strict interpretation of the code would confine our business announcement to a plain terse statement as to our name, business, location and office hours, and that the sign above the door should be modest and neat; and that all or any demonstration, either by black or white, gilt or gold, that savor or smack of individual superiority, would be reprehensible. That this is the intention of the code, its animus or spirit, I think we all admit.

In business circles the use of printer's ink, showy and attractive windows, the hand-bills and dodgers, electrical display, signs, etc., are all admitted as being perfectly clean and regular in attracting business, and no difference how unworthy the promoter, or how inferior the goods, yet, if he succeeds, he is hailed as sharp, shrewd and progressive.

In our own profession, no difference how skillful, careful and conscientious one may be, no matter, if as a dentist, he may be able to do better work, render superior services, and at a less fee than his competitors, yet he must perforce keep out of the papers, cut down his sign and wait. The victim may know that his successful competitor is a poor workman, that he is ignorant, vulgar and conscienceless, yet he does not enlighten the public as to his own superior qualities at the risk of losing professional caste. To go even further into the matter, a claimant for business must not do these unprofessional things even at the expense of ultimate failure.

Now what makes a standard of right for selling the commodities of life, the transferring of property, the supplying of artificial limbs and eyes, the investing of money, the burying of the dead, and a separate standard for healing the wounded, caring for the sick and the making of artificial teeth ?

In tracing back to the genesis of the code, we do not find that advertising is recognized as being in the abstract inherently wrong, but that the hitch comes in the manner in which we may or might make our business announcement. This premise having been admitted, I now come to the meat of the subject which includes some of the different methods of giving notice of our individual claims to patronage. It has been my fortune to come in close contact with some of the most persistent advocates of a strict interpretation of the code, and I have usually found them with a business to protect, and prompted by this thought, I have been led to notice how these and other dentists "give notice" and still keep within the pale of the law, or, to dispense with the metaphor, keep under cover of the code.

Now let us remember and keep in mind that the thing we are trying to do, in maintaining the code, is to repress and prevent the making of special claims to superiority over our fellow-practitioners, and that it is the manner in which we do it, that "fires the patriotic heart," and I cite some of the common advertising tricks not covered by the code.

Probably one of the commonest, most contemptible means employed to hoist and bolster one's self, is the speaking of a competitor in a deprecatory manner, a suggestive shrug of the shoulder, the elevating of the eyebrows, and a covert sneer at his work or mention of his name.

One of the silly and amusing, though none the less effective methods of dodging the code, is the "Professor" racket. It has been my luck to meet several of these gentlemen who have risen to the high plane of demonstrator in a dental school, and who have borne out into the world the proud title of "Professor." Even the real professors in some of our schools have profited by their places and have ever after hated most heartily the newspaper "ad."

The horror at a loud sign, of a gentleman I have in mind, who advertised through his being the dean of a dental school, was only equaled by the pains he took to let the world know that he was a dean.

In the society columns of the Sunday papers you will occasionally see a notice reading something like this: Dr. So-and-So, whose dental parlors are a dream of luxury, whose equipments are second to none, whose clientile is made up of the first families in the city, has gone to take a much needed rest, etc. And then again we have the fraternity man who plays his lodge for all it's worth. A close second to him is the *bright* young dentist who joins the leading church, and who attends all the means of grace with a pocketful of his cards.

Recently, a new one to me, was the man who was invited to a social affair, and to each person to whom he was introduced, he volunteered the information that he was a graduate of the only school in America recognized in Europe. Still another dodge is the dentist who has an "Opening," and I read you a literal write-up: "The new dental parlors of Dr. Justout, on the third floor of Curvature's Hall, are models of comfort and elegance, than which there are no handsomer ones in any respect in this city. These parlors are tastily and beautifully decorated, wellappointed and perfectly equipped. The reception parlor is in every way a distinctive attraction as to decorations. This suite of parlors possesses every advantage of a modern and up-to-date establishment." This is advertising that reeks with braggadocio, smacks with superiority loud and blatant, pretentious and untrue.

How nauseating those words "dental parlor" have become, for we have the chiropodist parlor, the tonsorial parlor and the dental parlor tastily decorated, models of comfort and luxury, second to none, etc. These have been given to us in the "Sunday Jolly," until the words have become as much of a nightmare to us as Twain's "Punch, brothers punch, punch with care, punch in the presence of the passengair."

Recently a patient suggested to me that it was strange that in the State and District societies Dr. Pumpwind was the only recognized dentist from his town, that he, the doctor, informed him that his brother practitioners were not in good form professionally. This dentist would very well be described in the words of Carlyle, "Wi' his mouth full of wind, and his belly full of dust."

Now the intention of all these gentlemen is to advertise and still avoid violating the letter, not the spirit of the code, and this purpose is none the less plain because of their assuming to be correct professionally. That there is a moral and legal right to advertise one's business can not be gainsaid, but that the violation of the spirit of the code by these various subterfuges and shades of deceit can ever be called right, manly or just, I most emphatically deny. We have a slang phrase which has almost become proper by common use, that applies to so many of the profession, namely, the "knocker," a quiet, well-directed little tap that is effectual in hurting the competitor and boosting the knocker.

As for myself, I rather prefer the independent free lance who uses the poster, the dodger, flash-lights and the newspaper columns, and all of the open methods of advertising, to the coward, hypocrite and sneak. The one is a fair outspoken hustler for business, while the other is a skulker and a coward. Brought to its final issue, looked at in a reasonable and fair way, laying aside the cloak of the profession, and meeting your competitor as other callings meet theirs, we must admit that much of the non-advertising spirit is humbug, snobbery and worst of all, cowardice and selfishness.

Advertising must be either right or wrong, and I have yet to hear any intelligent reason offered why the devil may trumpet his claims to goodness, and the angels be condemned to silence. There are some souls who have climbed to the astral plane, who maintain that all advertising is wrong, but the world does not accept that view of the matter, and as long as the conditions of life continue as they are, as long as the struggle for existence goes on, just so long will we have a race, a people and a business world that will be determined and shaped by the law of the survival of the fittest.

Utility is the ultimate appeal on all questions of ethics. The value of material things is determined by their usefulness—a law, a code, a constitution, are men-made things, and when they are insufficient "to make the punishment fit the crime," when they do not meet the exigencies of the case their sacredness falls from them, they are extraneous and like old clothes that are worn and soiled, they are to be cast aside. The wheels of progress are moving forward in even and definite lines, and the ultimate is the all of life, and our profession owes to itself and posterity the fostering of the spirit of fairness, breadth of thought, liberality of sentiment and freedom of action. If all of the members of the profession were imbued with the spirit of fairness, if we were all unselfish and just, if we could even coerce our fellows into these conditions, there might be some hope for the code of ethics; but as long as our colleges and the profession admit the class of men they do, to the ranks of the profession, and as long as the *smart* and *sharp* people are allowed to take precedence over strength, character, worth and quality in other men, as long as the hypocrite, the shoddy and snobbish pretender can find a field to operate in, just so long will the delectable mountains of a code-regulated profession be always a dream of the future, a lost golden thread and the ever-deluding mirage of professional fairness.

